# Significance of Circulating Tumor Cells Detected by the CellSearch System in Patients with Metastatic Breast Colorectal and Prostate Cancer

**DOI:** 10.1155/2010/617421

**Published:** 2009-12-09

**Authors:** M. Craig Miller, Gerald V. Doyle, Leon W. M. M Terstappen

**Affiliations:** ^1^Veridex LLC, Huntingdon Valley, PA 19006, USA; ^2^Department of Medical Cell BioPhysics, MIRA Institute for Biomedical Technology and Technical Medicine, Faculty of Science and Technology, University of Twente, Drienerlolaan 5, 7500AE Enschede, The Netherlands

## Abstract

The increasing number of treatment options for patients with metastatic carcinomas has created a concomitant need for new methods to monitor their use. Ideally, these modalities would be noninvasive, be independent of treatment, and provide quantitative real-time analysis of tumor activity in a variety of carcinomas. Assessment of circulating tumor cells (CTCs) shed into the blood during metastasis may satisfy this need. We developed the CellSearch System to enumerate CTC from 7.5 mL of venous blood. In this review we compare the outcomes from three prospective multicenter studies investigating the use of CTC to monitor patients undergoing treatment for metastatic breast (MBC), colorectal (MCRC), or prostate cancer (MPC) and review the CTC definition used in these studies. Evaluation of CTC at anytime during the course of disease allows assessment of patient prognosis and is predictive of overall survival.

## 1. Introduction

In 1869 Thomas Ashworth, an Australian physician, after observing microscopically circulating tumor cells in the blood of a man with metastatic cancer, postulated that “*…*. cells identical with those of the cancer itself being seen in the blood may tend to throw some light upon the mode of origin of multiple tumours existing in the same person.” After comparing the morphology of the circulating cells to tumor cells from different lesions Ashworth concluded that “One thing is certain, that if they [CTC] came from an existing cancer structure, they must have passed through the greater part of the circulatory system to have arrived at the internal saphena vein of the sound leg” [1]. Over the succeeding one hundred and forty years, cancer research has indeed demonstrated the critical role circulating tumor cells play in the metastatic spread of carcinomas [2]. However, it has only been shown recently—after development of technologies with the requisite sensitivity and reproducibility—that the diagnostic potential of these rare cells could be exploited [3–7].

To date, a variety of research methods have been developed to isolate and enumerate CTC [8]. The CellSearch Circulating Tumor Cell System has been validated via a rigorous clinical testing program [9–18] and is the only FDA cleared device for the enumeration of CTC in whole blood. The significance of the CellSearch assay results has been amply demonstrated, first in an extensive pilot study program and then in a series of prospective, well-controlled pivotal clinical trials. It is the agreement seen between CTC levels in patients with metastatic breast, colorectal, or prostate cancer and the true clinical endpoint overall survival that we will review.

## 2. Materials and Methods

### 2.1. Study Design

Three prospective trials were conducted in clinical centers throughout the United States, The Netherlands, and the UK to evaluate the ability of CTC to predict PFS (Progression Free Survival) and OS (Overall Survival) in patients with MBC, MCRC, and MPC cancer. The principal inclusion criteria for the MBC and MCRC trials were measurable disease, while all patients in the MPC study had castration resistant disease defined as two consecutive increases in PSA despite standard hormonal management. Patients commencing a new first- or second-line cytotoxic therapy for MPC or MCRC and any new line of therapy for MBC were eligible. All patients had an ECOG performance status score of 0 to 2 and hemoglobin ≥8 g/dL. Patients with brain metastases or a history of other malignancies within the last five years were excluded.

All patients were enrolled on an Intent-to-Treat basis. The institutional review boards at each center approved the study protocol and all patients provided written informed consent. The CellSearch system was used to enumerate CTC in 177 MBC, 430 MCRC, and 231 MPC patients, respectively. 7.5 mL of blood was drawn from patients before starting a new line of therapy and at monthly intervals after initiation of therapy [10–16]. An independent clinical research organization collected and monitored all clinical and laboratory data. Patients were followed up with regular chart review for up to 36 months from the time of the baseline blood draw for the survival analysis. For post-treatment samples, the measurement was taken from the time of the sample to avoid lead time bias. Patients were categorized prospectively as having either Unfavorable or Favorable CTC counts. For MBC and MPC, Unfavorable CTC were defined as ≥5 CTC/7.5 mL and for MCR ≥3 CTC/7.5 mL.

### 2.2. Enumeration of Circulating Tumor Cells

Blood samples were drawn into 10 mL evacuated blood draw tubes (CellSave, Veridex, Raritan, NJ), maintained at room temperature, and processed within 96 hours of collection. The evaluations were performed in a blinded fashion in one of four central laboratories (Veridex LLC, Huntingdon Valley, PA; Veridex LLC, Enschede, The Netherlands; IMPATH Predictive Oncology, Los Angeles, CA; and Cleveland Clinic, Cleveland, OH). The CellSearch System (Veridex LLC, Raritan, NJ) consists of the CellTracks Autoprep, CellTracks Magnest, CellSearch Epithelial Cell Kit, and the CellTracks Analyzer II. The CellSearch Epithelial Cell Kit contains ferrofluids labeled with the epithelial cell adhesion molecule (EpCAM), the staining reagents 4′,2-diamidino-2-phenylindole, dihydrochloride (DAPI), CD45-Allophycocyan (CD45-APC), and cytokeratin 8,18 Phycoerythrin and cytokeratin 19 Phycoerythrin (CK-PE), buffers to enhance the cell capture [19, 20], and permeabilizes and fixes the cells.

The CellTracks Autoprep immunomagnetically enriches cells expressing EpCAM from 7.5 mL of blood, fluorescently labels the enriched cells with DAPI, CD45-APC, and CK-PE, and resuspends the cells in the cartridge placed in the CellTracks Magnest. The design of the magnets guides the magnetically labeled cells to the analysis surface [21] and the CellTracks Magnest containing the chamber is placed on the CellTracks Analyzer II. This semiautomated fluorescence-based microscopy system acquires images using a 10X NA0.45 objective with filters for DAPI, FITC (not used), PE and APC to cover the complete surface area of the analysis chamber. A computer identifies objects staining with DAPI and PE in the same location and generates images for the DAPI, FITC, PE, and APC filters. A reviewer selects the CTC defined as nucleated DAPI+ cells, lacking CD45 and expressing CK-PE from the gallery of objects which are tabulated by the computer. Accuracy, precision, linearity, and reproducibility of the CellSearch have been described elsewhere [17].

### 2.3. Statistical Analysis

For this study Kaplan-Meier plots were generated based on CTC at baseline and follow-up blood collections for survival analyses. Survival curves were compared using log-rank testing. Cox proportional hazards regression was used to determine univariate and multivariate hazards ratios for overall survival.

## 3. Results

### 3.1. Classification of Circulating Tumor Cells

**Figure 1:**
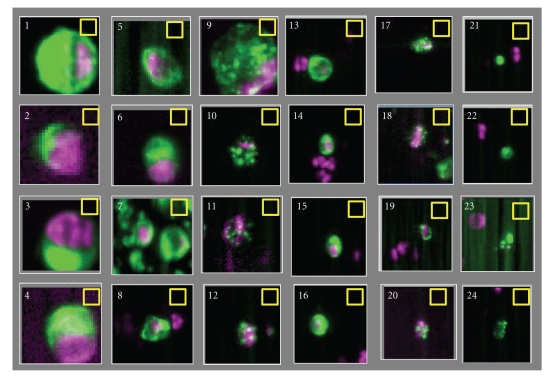
Images of twenty-four CTC candidates identified by the CellTracks Analyzer II. The images show an overlay of DAPI purple and CK-PE green. No staining was observed in the CD45 APC and FITC channel at the position of the CTC candidates. Six operators trained to review CellSearch CTC data classified objects 1–10, 13–17 as CTC, and discard objects 20–24 as CTC. 1 of 6 operators classified object 11 as a CTC and 5 of 6 operators classified object 12, 18, and 19 as a CTC.

Definitions of CTC vary widely between studies and have lead to a large range of reported CTC frequencies. CellSearch provides a highly standardized and automated platform to detect tumor cells in whole blood. In the CellSearch system final classification of CTC is performed by the operator and is the main contributor of the error of the assay [22]. Consistency in CTC classification among operators is therefore important. To illustrate that this can be challenging is illustrated by the images of 24 CTC candidates in Figure 1. Green represents the CK-PE staining and purple the DAPI staining. The corresponding APC and FITC images of the 24 candidates showed no staining. Only objects that are larger in size than the 4 × 4 *μ*m yellow boxes can be classified by the operators as CTC. Most operators will have no problems assigning objects 1–8 as CTC and will discard objects 21–24. The latter is presented to the operator because of the presence of DAPI in the vicinity of a CK-PE positive object. Disagreement can occur when objects 9–20 are classified. Six operators that classified CTC in the three prospective multicenter studies were asked to classify the objects in Figure 1. The granulated objects in frames 9–12 clearly show features consistent with apoptosis [23]. All operators classified objects 9 and 10 as a CTC, 1 of 6 classified objects 11 as a CTC, and 5 of 6 classified objects 12 as a CTC. The small objects 13–16 were classified as CTC by all operators. Similar to the objects 9–12, objects 17–20 show the granulated CK-PE only; now the objects are smaller. All operators classified object 17 as a CTC, none classified object 20 as a CTC, and 5 of 6 classified objects 18 and 19 as a CTC.

### 3.2. Frequency of Circulating Tumor Cells in Cancer Patients and Controls

The frequency of CTC in 7.5 mL of blood from 295 normal donors, 255 patients with benign disease, and 177 MRC, 413 CRC, and 218 MPC patients before initiation of a new line therapy is shown in Table 1. Only a small fraction of the objects presented by the computer to the operator are classified as CTC. This is illustrated in Table 2 showing the average, standard deviation, and median number of events detected by the software as CK+ and DAPI+ in blood from normal donors, patients with benign disease, MBC, MCRC, and MPC patients with 0 CTC, 1–4 CTC, and 5 or more CTC in 7.5 mL of blood.

### 3.3. Relation between the Presence of CTC and Overall Survival (OS) in MBC, MCRC, and MPC

**Figure 2:**
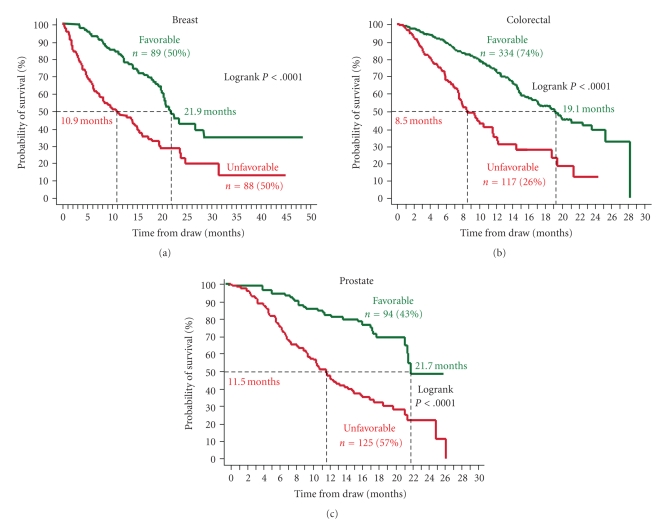
Kaplan Meier Analysis of overall survival before starting a new line of therapy for patients with metastatic breast cancer (a), metastatic colorectal cancer (b), and castration resistant prostate cancer (c). Patients were divided into those with Favorable and Unfavorable CTC. The cutoff value between favorable and unfavorable CTC was ≥5 CTC/7.5 mL blood for breast and prostate cancer and ≥3 CTC/7.5 mL blood for prostate cancer.

For MBC and MPC a threshold of 5 CTC/7.5 mL and for MCRC a threshold of 3 CTC/7.5 mL were used to stratify patients into those with Favorable outcomes (CTC <3 or <5) and those with Unfavorable outcomes (CTC ≥3 or ≥5). The Kaplan Meier curves in Figure 2 show the probability of OS for patients with Favorable and Unfavorable CTC counts before the initiation of therapy. Median OS for MBC, MCRC, and MPC patients with Favorable CTC was 21.9 (95% CI: 20.1–28.6), 18.5 (95% CI: 15.2–21.2), and 21.7 (95% CI: 21.3–NR) months, respectively. In contrast median OS for MBC, MCRC, and MPC patients with Unfavorable CTC was 10.9 (95% CI: 7.0–15.2), 9.4(95% CI: 7.5–11.6), and 11.5 (95% CI: 9.3–13.7) months, respectively. The differences are highly significant with logrank *P*-values <.0001.

**Figure 3:**
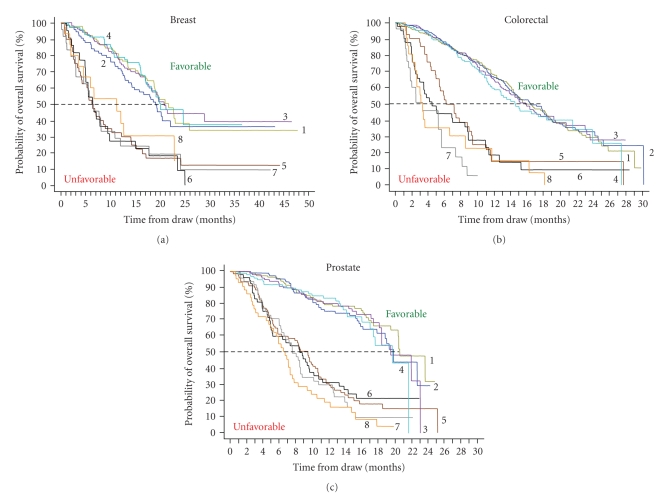
Kaplan Meier Analysis of overall survival. Favorable CTCs are indicated with 1 through 4 and Unfavorable CTCs with 5 through 8. (a) Median overall survival of MBC patients with Favorable CTC after 3–5 Weeks (1) (*n* = 92), 6–8 Weeks (2) (*n* = 77), 9–14 Weeks (3) (*n* = 105) and 15–20 Weeks (4) (*n* = 70) of treatment median was 21.7, 19.1, 20.8, and 20.1 months, respectively. Median overall survival of MBC patients with Unfavorable CTC after 3–5 Weeks (5) (*n* = 40), 6–8 Weeks (6) (*n* = 22) 9–14 Weeks (7) (*n* = 24), and 15–20 Weeks (8) (*n* = 15) of treatment was 6.2, 6.3, 6.4, and 11.3 months, respectively. (b) Median overall survival of MCRC patients with Favorable CTC after 1–2 Weeks (1) (*n* = 316), 3–5 Weeks (2) (*n* = 292), 6–12 Weeks (3) (*n* = 285), and 13–20 Weeks (4) (*n* = 172) of treatment was 15.7, 16.4, 15.8, and 14.6 months, respectively. Median overall survival of MCRC patients with Unfavorable CTC after 1–2 Weeks (5) (*n* = 41) 3–5 Weeks (6) (*n* = 41), 6–12 Weeks (7) (*n* = 25), and 13–20 Weeks (8) (*n* = 21) of treatment median was 6.1, 4.4, 3.3, and 3.3 months, respectively. (c) Median overall survival of MPC patients with Favorable CTC after 2–5 Weeks (1) (*n* = 123), 6–8 Weeks (2) (*n* = 110), 9–12 Weeks (3) (*n* = 100), and 13–20 Weeks (4) (*n* = 99) of treatment median was 20.7, 19.9, 19.6, and 19.8 months, respectively. Median overall survival of MPC patients with Unfavorable CTC after 2–5 Weeks (5) (*n* = 80), 6–8 Weeks (6) (*n* = 53), 9–12 Weeks (7) (*n* = 49), and 13–20 Weeks (8) (*n* = 44) of treatment was 9.5, 8.5, 7.6, and 6.7 months, respectively. The cutoff value between favorable and unfavorable CTC was ≥5 CTC/7.5 mL blood for breast and prostate cancer and ≥3 CTC/7.5 mL blood for prostate cancer.

Figure 3 shows the Kaplan Meier curves of the probability of OS for patients with Favorable and Unfavorable CTC counts at monthly intervals after initiation of therapy. At all time points tested the difference in survival between the Favorable and Unfavorable groups is highly significant (logrank *P*-values ≤.0070). CTCs thus predict outcomes at any time during treatment and can be used to monitor treatment.

**Figure 4:**
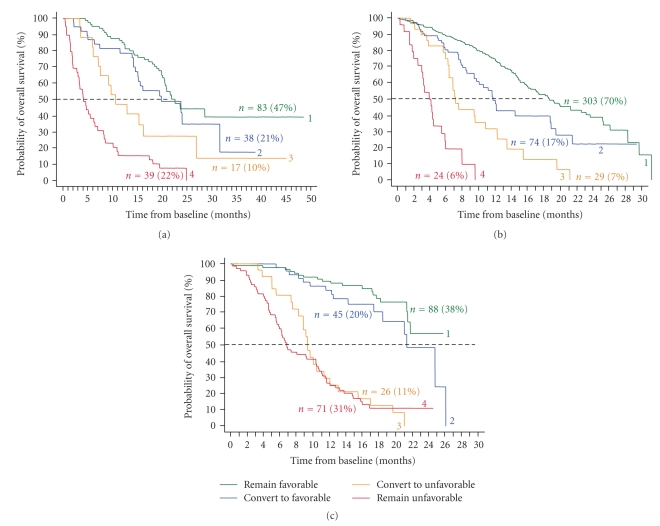
CTC changes after treatment in patients with MBC (a), MCRC (b), and MPC (c). Curves labeled 1 represent patients with CTCs that remain Favorable, labeled 2 CTCs convert from Unfavorable to Favorable, labeled 3 CTCs convert from Favorable to Unfavorable, and labeled 4 CTC remain Unfavorable. (a) After initiation of therapy, CTC in 83 (47%) MBC remained Favorable with median overall survival (OS) of 22.6 months, CTC in 39 (22%) patients remained Unfavorable median OS 4.1 months, CTC in 38 (21%) patients converted to Favorable CTC median OS 19.8 months, and CTC converted to Unfavorable in 17 (10%) patients median OS 10.6 months. (b) CTC in 303 (70%) MCRC remained Favorable median OS 18.6 months, CTC in 24 (6%) patients remained Unfavorable median OS 3.9 months, CTC in 74 (17%) patients converted to Favorable CTC median OS 11.7 months, and CTC converted to Unfavorable in 29 (7%) patients median OS 7.1 months. (c) CTC in 88 (38%) MPC remained Favorable median OS of more than 26 months, CTC in 71 (31%) patients remained Unfavorable median OS 6.8 months, CTC in 45 (20%) patients converted to Favorable CTC median OS 21.3 months, and CTC converted to Unfavorable in 26 (11%) patients median OS 9.3 months. The cutoff value between favorable and unfavorable CTC was ≥5 CTC/7.5 mL blood for breast and prostate cancer and ≥3 CTC/7.5 mL blood for prostate cancer.

To answer whether or not a change in CTC “after” treatment can alter survival prospects, further Kaplan Meier analyses were performed and shown in Figure 4. Patients were divided into four groups: (1) CTCs remain Favorable (green lines); (2) CTCs remain Unfavorable (red lines); (3) CTCs change from Favorable to Unfavorable (orange lines); (4) CTCs change from Unfavorable to Favorable during the course of therapy (blue lines). In all three cancers, patients with persistent CTC counts had the worst outcome and strongly suggested that they were on a futile therapy. Also patients that develop CTC during the course of therapy convert to a poor prognosis, similar to those with Unfavorable CTC before and after therapy. In contrast patient's prognosis improved for those converting from the Unfavorable to the Favorable group. This was the strongest for MPC.

In both univariate and multivariate analysis CTC levels at all time points were significantly associated with overall survival [10, 13, 15].

## 4. Discussion

In most cases death from cancer is not caused by expansion of the primary tumor, but through dissemination of the disease. To settle in distant sites tumor cells must travel through the peripheral blood; question is at what frequency. In routine analysis of 10^4^ leukocytes (∼1 *μ*L of blood) tumor cells are not observed. Using immunomagnetic enrichment from 20 mL of blood followed by flowcytometric analysis we were able to identify CTCs in blood from most carcinoma patients [24, 25].

To minimize error in CTC enumeration the sample preparation steps needed to be automated. A blood volume of 7.5 mL was chosen for the development of the standardized CellSearch CTC enumeration system as this could be routinely obtained from a 10 mL draw tube. The automated system utilizes immunomagnetic nanoparticles directed against the epithelial cell adhesion molecule (EpCAM) to isolate and concentrate epithelial tumor cells [17, 19]. Once enriched, the cells are identified using epithelial cell specific fluorescently labeled anticytokeratin (CK) monoclonal antibodies, the nuclear stain (DAPI), and absence of the pan-leukocyte counter stain (CD45-APC). It should be noted that assay success is dependent upon the level of expression of the EpCAM and Cytokeratin target antigens which can vary significantly [8]. We have investigated these issues and have demonstrated that although antigen expression varies considerable, the assay is capable of functioning within a wide range of EpCAM and cytokeratin levels [19, 20].

To reduce the background observed in blood from healthy donors flowcytometry used in our original work [24, 25] was replaced by fluorescence microscopy. The addition of morphology to the CTC definition indeed increased the specificity but was accompanied by a reduction of the CTC frequency in cancer patients. In model systems 1 tumor cell spiked into 7.5 mL of blood containing ∼5 × 10^7^ leukocytes and 4 × 10^10^ erythrocytes can be identified and recovery of spiked tumor cells is ∼80% [17, 19, 20]. Whereas the morphology of cells from tumor cell lines is relatively homogenous, a large heterogeneity is observed in tumor cells in blood from cancer patients. This heterogeneity introduces variation in the assignment of EpCAM+, Cytokeratin 8,18, or 19, CD45−, DAPI+ objects as CTC by the operator [22]. The most likely explanation for the large variation in the reported frequency of CTC by different methods using a similar phenotypic definition is the differences in the actual definition of a CTC [23–30]. That less stringent criterion for CTCs results in a significant increase in the reported numbers as illustrated in Table 2 showing the number of EpCAM enriched CK+DAPI+ events for patients with zero, 1–4, and 5 or more CTC. The CellSearch definition of a CTC was set before the initiation and validated by prospective clinical studies in MBC, MCRP, and MPC. Further studies will be needed to determine the value, if any, of the CK+ DAPI+ events detected that were not classified as CTC.

The prospective multicenter studies in MBC, MCRC, and MPC demonstrated that presence of CTC was a strong predictor of poor outcome; see Figure 2. Even after a few weeks of therapy, patients with Unfavorable CTC had much shorter overall survival than did patients with Favorable CTC; see Figure 3. Effective therapies—ones that result in elimination of CTC—can prolong survival, irrespective of the line of therapy, as shown by the improvement in overall survival in patients with MBC, MCRC, and MPC that converted from an Unfavorable to Favorable CTC count; see Figure 4. This benefit was seen in patients independent of line of therapy, indicating that treatment can still be effective in patients that have failed a previous therapy. Today, the current standard of care calls for routine assessments of a patient's clinical status at or about one-month intervals depending on the type of therapy. Imaging studies are an exception as they are usually performed at some intermediate time point and at the end of a given line of therapy. Consequently most clinicians do not entertain a change in treatment until after several cycles of drug have been administered. It is also felt that a certain minimum time, usually two to three cycles of therapy is often needed before clinical benefit may be evident. Thus the advantages associated with changing treatment at a significantly earlier time point must be demonstrated.

In this review we only addressed the clinical relevance of CTC detected in patients with metastatic disease. Although CTC can also be detected with the CellSearch in the adjuvant setting and primary setting albeit at a lower frequency, no prospective clinical trials have been concluded to determine its clinical relevance [31].

## 5. Conclusions

CellSearch is a validated system to enumerate Circulating Tumor Cells in 7.5 mL of blood. In prospective multicenter studies in metastatic breast, colorectal, and prostate cancer the presence of CTC before and after initiation of a new line of therapy was the strongest predictor of poor overall survival.

## Figures and Tables

**Table 1:** Frequency of CTC in 7.5 mL of blood from normal donors: patients with benign disease and metastatic breast, colorectal, and prostate cancer patients before initiation of a new line therapy.

	Percentage of patients with CTC above threshold							
	# pat	≥1	≥5	≥10	≥50	≥100	≥500	≥1000
Normal	295	3.4	0.0	0.0	0.0	0.0	0.0	0.0
Benign	255	7.5	0.4	0.4	0.0	0.0	0.0	0.0
Breast	177	70.6	49.7	38.4	20.9	15.8	3.4	2.8
Colorectal	413	47.5	18.2	11.6	2.4	1.0	0.0	0.0
Prostate	218	77.5	57.3	45.0	20.6	13.8	3.7	2.3

**Table 2:** Frequency of events detected by the software as CK-PE+ and DAPI+ that were not classified as CTC in 7.5 mL of blood from normal donors: patients with benign disease, and metastatic breast, colorectal and prostate cancer patients before initiation of a new line therapy.

	CTC											
	0				≥1–<5				≥5			
	*n*	mean	SD	med	*n*	mean	SD	med	*n*	mean	SD	Med
Normal	285	53	68	40	10	81	108	42	0	—	—	—
Benign	236	38	30	30	18	44	26	47	1	159	—	159
Breast	50	53	29	29	36	73	93	38	86	403	1182	84
Colorectal	217	223	1070	78	121	266	468	116	75	603	1620	230
Prostate	49	420	1713	86	44	222	358	101	125	1301	3020	412
